# The Effect of a Rotating Magnetic Field on the Regenerative Potential of Platelets

**DOI:** 10.3390/ijms25073644

**Published:** 2024-03-25

**Authors:** Elżbieta Cecerska-Heryć, Małgorzata Goszka, Marta Gliźniewicz, Bartłomiej Grygorcewicz, Natalia Serwin, Patrycja Stodolak, Weronika Słodzińska, Radosław Birger, Aleksandra Polikowska, Marta Budkowska, Rafał Rakoczy, Barbara Dołęgowska

**Affiliations:** 1 Department of Laboratory Medicine, Pomeranian Medical University of Szczecin, Powstancow Wielkopolskich 72, 70-111 Szczecin, Poland; malgosia@goszka.pl (M.G.); marta.glizniewicz@pum.edu.pl (M.G.); natalia.serwin@pum.edu.pl (N.S.); patrycjastodolak23@gmail.com (P.S.); 63467@student.pum.edu.pl (W.S.); radoslawbirger@gmail.com (R.B.); polikowska.aleksandra@gmail.com (A.P.); barbara.dolegowska@pum.edu.pl (B.D.); 2 Department of Chemical and Process Engineering, West Pomeranian University of Technology, Piastów 42, 71-311 Szczecin, Poland; bartlomiej.grygorcewicz@pum.edu.pl (B.G.); rafal.rakoczy@zut.edu.pl (R.R.); 3 Department of Forensic Genetic, Pomeranian Medical University of Szczecin, Powstancow Wielkopolskich 72, 70-111 Szczecin, Poland; 4 Department of Medical Analytics, Pomeranian Medical University of Szczecin, Powstancow Wielkopolskich 72, 70-111 Szczecin, Poland; marta.budkowska@pum.edu.pl

**Keywords:** rotating magnetic field, IGF-1, PDGF-BB, TGF-β1, FGF-1

## Abstract

Platelets are actively involved in tissue injury site regeneration by producing a wide spectrum of platelet-derived growth factors such as PDGF (platelet-derived growth factor), IGF-1 (insulin-like growth factor), TGF-β1 (transforming growth factor β), FGF (fibroblast growth factor), etc. A rotating magnetic field (RMF) can regulate biological functions, including reduction or induction regarding inflammatory processes, cell differentiation, and gene expression, to determine the effect of an RMF on the regenerative potential of platelets. The study group consisted of 30 healthy female and male volunteers (n = 15), from which plasma was collected. A portion of the plasma was extracted and treated as an internal control group. Subsequent doses of plasma were exposed to RMF at different frequencies (25 and 50 Hz) for 1 and 3 h. Then, the concentrations of growth factors (IGF-1, PDGF-BB, TGF-β1, and FGF-1) were determined in the obtained material by the ELISA method. There were statistically significant differences in the PDGF-BB, TGF-β1, IGF-1, and FGF-1 concentrations between the analyzed groups. The highest concentration of PDGF-BB was observed in the samples placed in RMF for 1 h at 25 Hz. For TGF-β1, the highest concentrations were obtained in the samples exposed to RMF for 3 h at 25 Hz and 1 h at 50 Hz. The highest concentrations of IGF-1 and FGF-1 were shown in plasma placed in RMF for 3 h at 25 Hz. An RMF may increase the regenerative potential of platelets. It was noted that female platelets may respond more strongly to RMF than male platelets.

## 1. Introduction

Platelets (PLTs) are the minor morphotic elements of blood that have the ability to adhere to the damaged vessel wall and form aggregates. In addition to their role in thrombosis and hemostasis, PLTs are increasingly recognized as key elements in many pathophysiological processes, including inflammation and atherogenesis, tumor growth and metastasis, wound healing, angiogenesis, and host antimicrobial defense, affecting both innate and acquired immune response [1,2].

In physiological conditions, circulating platelets in the blood are inactive and do not have the ability to form aggregates [3]. Platelet activation occurs through their adhesion to the extracellular matrix and the influence of agonists such as adenosine diphosphate (ADP), thromboxane A2 (TXA2), platelet-activating factor (PAF), cathepsin G, serotonin, and thrombin, which is the most potent physiological platelet agonist. These substances bind to membrane receptors, triggering a platelet response [4,5].

Platelet activation plays a crucial role in regulating the levels of various growth factors, and this process has significant implications for both health and disease. Platelet alpha granules contain numerous proteins, chemokines, cytokines, and growth factors (GFs) such as PDGF—platelet-derived growth factor, IGF—insulin-like growth factor, EGF—epidermal growth factor, HGF—hepatocyte growth factor, TGF-β—transforming growth factor β, FGF—fibroblast growth factor, and VEGF—vascular endothelial growth factor, which are released during platelet activation.

Growth factors affect all stages of the complex wound-healing process. They regulate the acquisition, growth, and differentiation of cells involved in the reconstruction of tissues, blood vessels and bones, angiogenesis, inflammatory processes, and blood coagulation. Any changes related to the reduction in the level of a specific GF, its receptors, or rapid degradation of growth factors may lead to delayed and abnormal wound healing, chronic inflammation, or fibrosis [6,7].

Furthermore, GFs modulate inflammation and recruit immune cells to the site of injury or infection. An imbalance in platelet activation and release of GFs may contribute to the autoimmune disorders or altered immune responses observed in certain diseases. The precise regulation of platelet activation and the subsequent modulation of GF levels are essential for maintaining health. Any dysregulation in this process can contribute to pathogenesis of various diseases, highlighting the importance of understanding and managing platelet function in a therapeutic context [8].

In recent years, scientific publications describing new possibilities and clinical applications of magnetic fields in medicine have constantly increased. Scientific studies demonstrate that magnetic fields can have a positive impact on the body.

Magnetic fields (MF) can be divided into different categories depending on frequency, intensity, or practical application parameters. There are static fields (SMF) and dynamic fields, which include pulsed magnetic fields (PMF), rotating magnetic fields (RMF), and sinusoidal magnetic fields. In terms of frequency, electromagnetic fields can be divided into very low (<300 Hz), low, medium, and high (30 kHz–30 MHz) [9,10,11].

RMF is a magnetic field where the opposite poles rotate around a central point. A rotating magnetic field arises as a resultant field during the superposition of two or more alternating current magnetic fields, having the same frequencies but spatially phased relative to each other [12]. RMF can be considered an electromagnetic stirrer. The use of this type of magnetic field involves inducing eddy currents in water media, which create their own magnetic field interacting with the primary field [13].

The biological effects of exposure to MF have been widely studied by many scientists at the cellular, tissue, and organ levels. Magnetic fields have been shown to have a beneficial effect on vasodilation, angiogenesis, accelerating repair, regeneration, and healing of soft tissues, nervous tissues and bones, analgesic aspects, anti-swelling, reducing inflammation and pain, and increasing oxygen flow to cells and tissues [10,14,15]. MF has been appreciated for its ability to modulate the metabolism of individual cells, increase or decrease cell proliferation, migration, and differentiation, stimulate enzyme systems [13,16,17,18,19,20,21], the immunomodulatory effect [22], and its impact on the level of reactive oxygen species (ROS) [11].

Electromagnetic field (EMF) stimulation is also being investigated in the context of the anticancer effects of various types of cancer, including skin, breast, prostate, lung, ovarian, pancreatic, thyroid, bladder, and colon cancers, both in vitro and in vivo [22,23,24,25,26,27]. It is worth noting that an electromagnetic field with the same parameters has a different effect on normal and cancerous cells. The biological effects of exposure to an EMF are related to its parameters and the physiological state of cells [28,29]. It can be concluded that EMFs have a selective cytotoxic effect on cancer cells [22,30,31,32].

Previous research focusing on the biological effects of an RMF has demonstrated its impact on reducing biofilm growth, bacterial biomass, and inhibiting the cellular activity of certain pathogens. This suggests that an RMF may act as an antiseptic for wounds [33]. A rotating magnetic field can also positively alter the activity of laccase, making it a promising tool for enhancing enzyme properties [13]. RMF exposure can increase resistance to heat stress, reduce levels of ROS, affect intracellular calcium ion concentrations, and contribute to cell aging deceleration [9,10].

Platelet-rich plasma and electromagnetic fields are widely used in various fields of medicine, in particular in regenerative medicine. In the scientific literature, one can find several studies evaluating the effect of magnetic fields on PRP and combining these two therapies to achieve better biological effects [34,35,36,37,38,39].

There are no reports in the literature regarding RMF’s impact on platelets’ regenerative capacity. However, taking into account the positive effect of electromagnetic fields, as well as the widespread use of PRP in various fields of medicine, the aim of our work was to check the potential effect of RMF on the production of platelet growth factors IGF-1, TGF-β1, PDGF-BB, and FGF-1 in blood plasma, depending on the frequency of RMF as well as the time spent in the field compared to an internal control group. The samples were exposed to a rotating magnetic field for 1 h and 3 h at frequencies of 25 and 50 Hz at a maximum induction of Bmax = 42.64 mT. Previous studies evaluating the biological effects of RMF have indicated that the listed RMF parameters produce the most effective results in modifying cellular metabolization activity, intracellular calcium ions, and ROS concentrations [9,13,33]. We deliberately used EDTA plasma instead of PRP to see if this easier-to-obtain material could become an alternative to PRP. We treat this study as preliminary research for further analyses comparing the amount of GFs in plasma and PRP under the influence of RMF to prove our hypothesis that an alternating magnetic field may stimulate platelets to produce platelet growth factors more than the centrifugation used in medical offices.

## 2. Results

### 2.1. Effect of a Rotating Magnetic Field on the Concentration of Platelet-Derived Growth Factor BB

Table 1 presents general descriptive statistics of the PDGF-BB concentrations [pg/mL] in the control and study groups exposed to rotating magnetic fields of various parameters (1 h 25 Hz, 1 h 50 Hz, 3 h 25 Hz, and 3 h 50 Hz).

**Figure 1:**
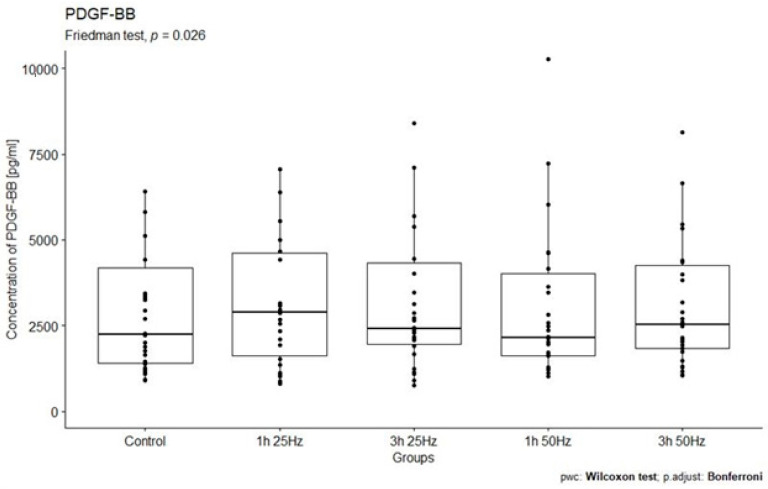
Comparison of PDGF-BB concentrations [pg/mL] among different groups. Friedman ANOVA analysis of the effect of the rotating magnetic field (RMF) on the PDGF-BB concentration [pg/mL] in plasma in study groups and the control group. Box–whisker plots with median. Intergroup differences in PDGF-BB concentration (*p* = 0.026).

The study analyzed the relationship between PDGF-BB concentrations in the study groups exposed to a rotating magnetic field (1 h 25 Hz, 1 h 50 Hz, 3 h 25 Hz, and 3 h 50 Hz) and the control group. A significant influence of the rotating magnetic field (RMF) on the concentration of PDGF-BB was demonstrated (*p* < 0.005; *p =* 0.026). The highest concentration of PDGF- BB was observed in samples placed in the RMF for 1 h at a frequency of 25 Hz (Figure 1).

### 2.2. Effect of a Rotating Magnetic Field on the Concentration of Transforming Growth Factor Beta 1

Table 2 presents general descriptive statistics of the TGF-β1 concentrations [pg/mL] in the control and study groups exposed to rotating magnetic fields of various parameters (1 h 25 Hz, 1 h 50 Hz, 3 h 25 Hz, and 3 h 50 Hz).

**Figure 2:**
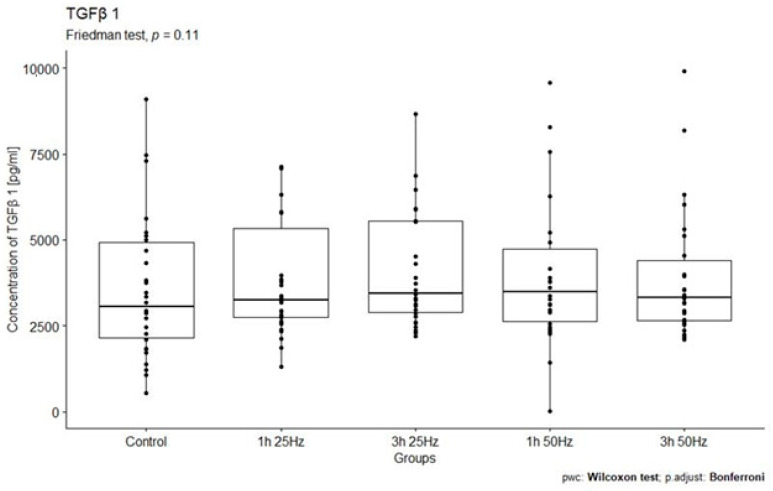
Comparison of TGF-β1 concentrations [pg/mL] among different groups. Friedman ANOVA analysis of the effect of the rotating magnetic field (RMF) on the TGF-β1 concentration [pg/mL] in plasma in study groups and the control group. Box–whisker plots with median. Intergroup differences in TGF-β1 concentration (*p =* statistically insignificant; *p* = 0.11).

The study analyzed no relationship between TGF-β1 concentrations in the study groups exposed to a rotating magnetic field (1 h 25 Hz, 1 h 50 Hz, 3 h 25 Hz, and 3 h 50 Hz) and the control group. The highest concentration of TGF-β1 was observed in samples placed in RMF for 3 h at 25 Hz and in samples subjected to field exposure for 1 h at 50 Hz (Figure 2).

### 2.3. The Influence of a Rotating Magnetic Field on the Concentration of Insulin-Derived Growth Factor 1

Table 3 presents general descriptive statistics of the IGF-1 concentrations [ng/mL] in the control and study groups exposed to RMFs of various parameters (1 h 25 Hz, 1 h 50 Hz, 3 h 25 Hz, and 3 h 50 Hz).

**Figure 3:**
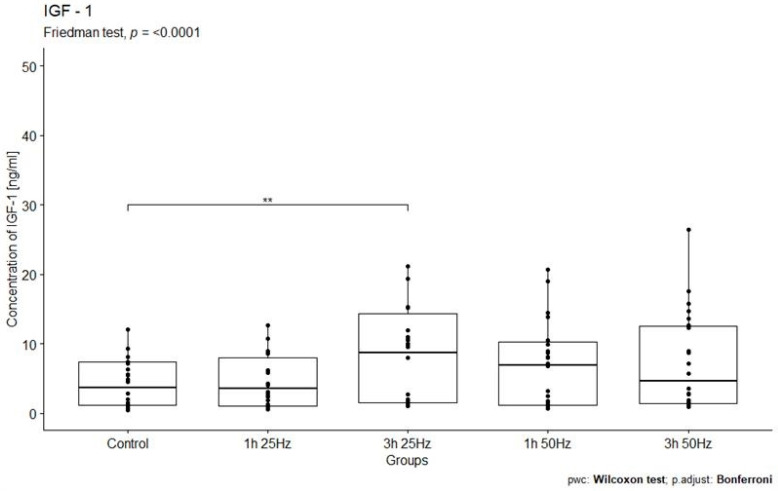
Comparison of IGF-1 concentrations [ng/mL] among different groups. Friedman ANOVA analysis of the effect of the rotating magnetic field (RMF) on the IGF-1 concentration [ng/mL] in plasma in study groups and the control group. Box–whisker plots with median. Intergroup differences in IGF-1 concentration (*p* < 0.0001). ** *p* < 0.01.

The study analyzed the relationship between IGF-1 concentrations in the study groups exposed to a rotating magnetic field (1 h 25 Hz, 1 h 50 Hz, 3 h 25 Hz, and 3 h 50 Hz) and the control group. A significant influence of the rotating magnetic field (RMF) on the concentration of IGF-1 was demonstrated (*p* < 0.0001). The highest concentration of IGF-1 was observed in samples placed in RMF for 3 h at 25 Hz (Figure 3).

### 2.4. The Influence of a Rotating Magnetic Field on the Concentration of Fibroblast Growth Factor 1

Table 4 presents general descriptive statistics of the FGF-1 concentrations [ng/mL] in the control and study groups exposed to RMFs of various parameters (1 h 25 Hz, 1 h 50 Hz, 3 h 25 Hz, and 3 h 50 Hz).

**Figure 4:**
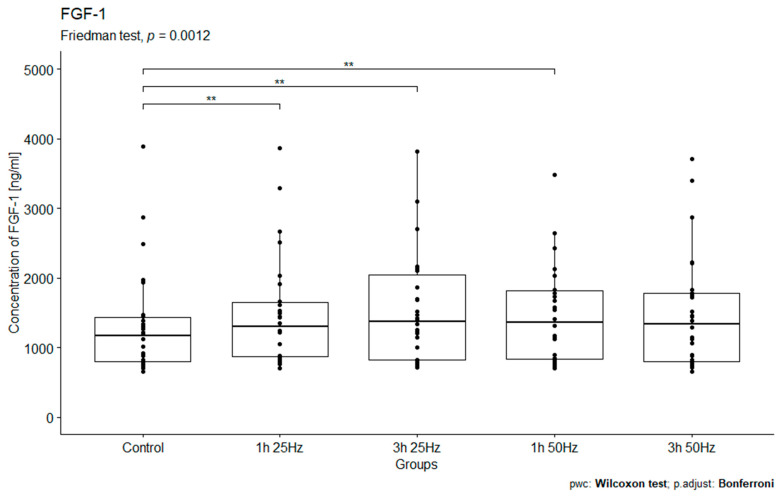
Comparison of FGF-1 concentrations [ng/mL] among different groups. Friedman ANOVA analysis of the effect of the rotating magnetic field (RMF) on the FGF-1 concentration [ng/mL] in plasma in the study and control groups. Box–whisker plots with median. Intergroup differences in FGF-1 concentration (*p =* 0.0012). ** *p* < 0.01.

The study analyzed the relationship between FGF-1 concentrations in the study groups exposed to a rotating magnetic field (1 h 25 Hz, 1 h 50 Hz, 3 h 25 Hz, and 3 h 50 Hz) and the control group. A significant influence of the rotating magnetic field (RMF) on the concentration of FGF-1 was demonstrated (*p* < 0.005; *p =* 0.0012). The highest concentration of FGF-1 was observed in samples placed in RMF for 3 h at 25 Hz and in samples subjected to field exposure for 1 h at 50 Hz (Figure 4).

### 2.5. The Influence of a Rotating Magnetic Field on the Concentration of Platelet Growth Factors Depending on Sex

**Figure 5:**
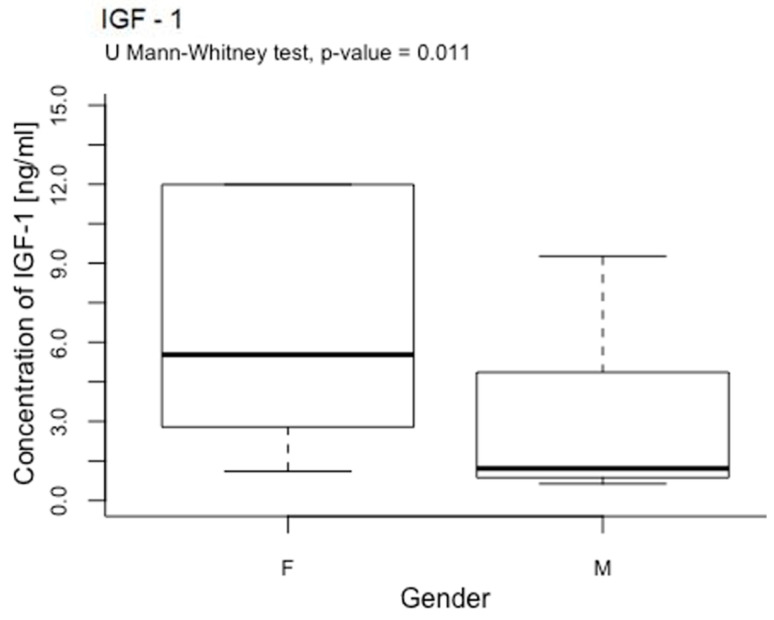
Comparison of IGF-1 concentrations [ng/mL] among women and men. U Mann–Whitney analysis of IGF-1 concentration [ng/mL] in plasma in women and men samples. Box–whisker plots with median. The difference in IGF-1 concentration between women and men (*p* < 0.05; *p* = 0.011).

**Figure 6:**
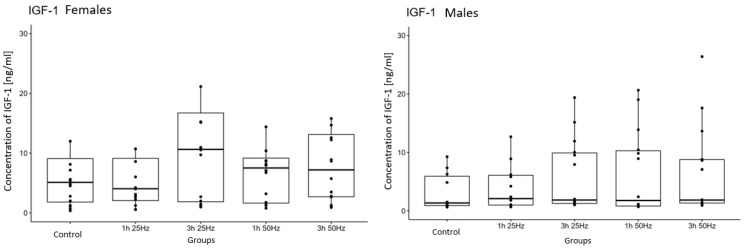
Comparison of changes in IGF-1 concentrations after exposure to RMF depending on the sex of volunteers. Box–whisker plots with median.

The study examined the effect of RMF on the concentrations of PDGF-BB, TGF-β1, IGF-1, and FGF-1 depending on the sex of the volunteers included in the study. A significant difference in IGF-1 concentration (*p* < 0.05; *p =* 0.011) was found between women and men after plasma exposure to a rotating magnetic field (Figure 5 and Figure 6). Higher concentrations of IGF-1 were obtained in the material collected from women in the case of other growth factors (PDGF-BB, TGF-β1, and FGF-1), and no significant differences were observed between women and men.

### 2.6. Platelet Concentrations in the Samples

Table 5 presents the general descriptive statistics of platelet concentrations in the samples for all cases, women and men. The concentration of platelets did not change after exposure of plasma to RMF.

The study examined the concentrations of platelets depending on the sex of the volunteers included in the study. No significant differences in platelet concentrations were observed between women and men (U Mann–Whitney test *p*-value = 0.14117).

Spearman’s rank correlation analysis was carried out between the examined platelet growth factors and platelet concentrations. No statistically significant correlations were found between the parameters.

### 2.7. The Strength of the Correlation between the Parameters

Spearman’s rank correlation analysis was carried out between the examined platelet growth factors and sex, age, height, and weight (control group and study groups) and the concentrations of biochemical parameters: cholesterol, HDL, LDL, triglycerides, glucose, albumin, iron, uric acid, creatinine, and protein total (control group). Statistically significant correlation results are presented in Table 6, Table 7, Table 8, Table 9, Table 10 and Table 11. Statistically significant negative weak correlations (in the study groups and the control group) were found between IGF-1 concentration and gender (Rs = −0.232), height (Rs = −0.188), weight (Rs = −0.257), between TGF-β1 concentration and weight (Rs = −0.270), between PDGF-BB concentration and height (Rs = −0.186) and weight (Rs = −0.180), and between FGF-1 concentration and height (Rs = −0.219). Positive low correlations were also observed between age and TGF-β1 concentration (Rs = 0.213), PDGF-BB concentration (Rs = 0.212), and between IGF-1 concentration and PDGF-BB concentration (Rs = 0.234). Positive moderate correlations were found between FGF-1 concentration and IGF-1 concentration (Rs = 0.464) and TGF-β1 concentration (Rs = 0.317). In addition, strong correlations were found between PDGF-BB concentration and TGF-β1 concentration (Rs = 0.648) and FGF-1 concentration (Rs = 0.564) and between FGF-1 concentration and age (Rs = 0.515). The values of the correlation coefficients are presented in Table 6.

Considering only the internal control group samples, a statistically significant negative moderately strong correlation between IGF-1 concentration and weight was found (Rs = −0.430). Positive correlations were also found between TGF-β1 concentration and PDGF-BB concentration (Rs = 0.696) and PDGF-BB concentration and FGF-1 concentration (Rs = 0.600). Positive moderately strong correlations were found between TGF-β1 concentration and FGF-1 concentration (Rs = 0.415) and between FGF-1 concentration and age (Rs = 0.498) and total protein concentration (Rs = 0.445). The values of the correlation coefficients are presented in Table 7.

In the study group exposed to RMF for 1 h at a frequency of 25 Hz, statistically significant positive moderately strong correlations were found between FGF-1 concentration and IGF-1 concentration (Rs = 0.426) and age (Rs = 0.475) and between TGF-β1 concentration and PDGF-BB concentration (Rs = 0.450). There was also a positive correlation between PDGF-BB concentration and FGF-1 concentration (Rs = 0.657). The values of the correlation coefficients are presented in Table 8.

In the study group exposed to RMF for 1 h at a frequency of 50 Hz, statistically significant positive strong correlations were found between the concentration of FGF-1 and the concentration of IGF-1 (Rs = 0.600) and the concentration of PDGF-BB (Rs = 0.600) and between the concentration of TGF-β1 and the concentration of PDGF-BB (Rs = 0.714). A positive moderately strong correlation was also found between FGF-1 concentration and age (Rs = 0.497). The values of the correlation coefficients are presented in Table 9.

In the study group exposed to RMF for 3 h at a frequency of 25 Hz, statistically significant positive strong correlations were found between FGF-1 concentration and IGF-1 concentration (Rs = 0.592), PDGF-BB concentration (Rs = 0.526) and age (Rs = 0.545), and between TGF-β1 concentration and PDGF-BB concentration (Rs = 0.519). The values of the correlation coefficients are presented in Table 10.

In the study group exposed to RMF for 3 h at 50 Hz, a statistically significant positive very strong correlation was demonstrated between the concentration of TGF-β1 and the PDGF-BB concentration (Rs = 0.818). There was also a positive strong correlation between FGF-1 concentration and age (Rs = 0.518) and IGF-1 concentration (Rs = 0.564), and a positive moderate strong correlation between FGF-1 concentration and TGF-β1 concentration (Rs = 0.387) and PDGF-BB concentration (Rs = 0.494). The values of the correlation coefficients are presented in Table 11.

### 2.8. Multi-Factor Evaluation of Relationships between the Tested Parameters

Multivariate regression analysis was performed. The influence of group, age, height, and weight (independent variables) on the concentrations of PDGF-BB, TGF-β1, IGF-1, and FGF-1 (dependent variables) was examined. In the case of IGF-1 and TGF-β1, it was shown that the increase in weight affected their concentration, causing a decrease in the amount by 0.26 ng/mL, respectively (0.09 µg/mL). In the PDGF-BB case, the entire model’s impact on its level was demonstrated to be 12%. Age increased the concentration of PDGF-BB by 0.19 pg/mL. In the case of FGF-1, the frequency and time spent in RMF, age, height, and weight affected the FGF-1 concentration by 18%. Higher age and height increased the FGF-1 concentration by 0.44 and 0.32 pg/mL (Table 12).

## 3. Discussion

Platelets regulate many physiological processes in the human body and are increasingly recognized as key elements in many pathophysiological processes. Active PLTs release numerous biologically active proteins from granules α, including growth factors such as PDGF, IGF, EGF, HGF, TGF-β, FGF, and VEGF [1,2,6,7,40,41]. The above study analyzed the effect of a rotating magnetic field on platelets’ regenerative potential. The influence of RMF frequency and plasma residence time in the field on the production of selected platelet growth factors IGF-1, TGF-β1, PDGF-BB, and FGF-1 was assessed.

Platelet-derived growth factor PDGF is a strong mitogen, a chemotactic factor for cells of mesenchymal or neuroectodermal origin, such as fibroblasts, vascular smooth muscle cells, glomerular mesangial cells, and grail cells of the brain, as well as monocytes and neutrophils. PDGF contributes to changes in cells’ shape and actin fibers’ reorganization; it is a strong vasoconstrictor [42]. It has mitogenic and chemotactic effects on osteoblasts, chondrocytes, and undifferentiated osteoprogenitor cells. PDGF plays an essential role at every stage of the healing process of soft tissues and bones [43].

Choi JH et al. [44], found increased expression of PDGF in human hair follicles exposed to an electromagnetic field with an intensity of 5–20 G and a frequency of 60 Hz. The PDGF mRNA expression levels progressively increased approximately 15-fold in the 5, 10, and 20 G electromagnetic field-treated HBS model compared to the control group.

Different results were obtained by Obayashi-Ishii M et al. [45]. They observed an inhibitory effect of a 150 mT static magnetic field on PDGF-AA secretion in human oral squamous cell carcinoma. They noted significantly reduced PDGF-AA levels by as much as 50% in the group subjected to the magnetic field (758 pg/mL) compared to the control group (1553 pg/mL). These results indicate that exposure to a magnetic field inhibited the production of PDGF-AA by tumor cell culture. In the case of cancer cells, suppression of PDGF-AA is beneficial because this factor is an autocrine regulator of VEGF that is involved in angiogenesis and tumor metastasis. The reasons for the contradictory results of the research may be differences in the type of applied field and the phenomenon of exerting the different influences of magnetic fields on normal and cancerous cells. The biological effects of exposure to electromagnetic fields are related to the parameters and the physiological state of cells [28,29].

Our study observed a significant impact of the rotating magnetic field on the concentration of PDGF-BB (*p* = 0.026) in plasma. The highest concentration of platelet-derived growth factor-BB was observed in the samples placed in the RMF for 1 h at a frequency of 25 Hz. Thus, the exposure of plasma to an RMF for 1 h at a frequency of 25 Hz significantly increases the release of platelet-derived growth factor-BB and the regenerative potential of platelets. These results also confirm those obtained by Choi et al. However, it should be noted that Choi used different electromagnetic field parameters than we did. Moreover, compared to those obtained by other scientists, our results indicate that, when using an RMF, the purpose of its use is very important. In healthy people, increasing PDGF activity is beneficial, but, in the case of cancer, it is not. Therefore, it is also important to know the impact of various RFM parameters on GF production.

Transforming growth factor beta TGF-β plays an important role in wound healing, inflammation, angiogenesis, re-epithelialization, and regeneration of connective tissue [46,47]. It regulates cell proliferation, migration, differentiation, and ECM production and modulates the immune response [48,49]. TGF-β ligands have a strong growth inhibitory effect in most cell types; an antiproliferative response can be observed in epithelial, endothelial, hematopoietic, and glial cells. TGF-β may also promote the proliferation of chondrocytes, osteoblasts, mesenchymal stem cells, some fibroblasts, and endothelial cells under certain conditions. TGFβ affects some key events in the physiology and proper development of organs, and disorders of its signaling occur in the pathogenesis of diseases such as connective tissue disorders, fibrosis, and cancer [50].

Various studies indicate that electromagnetic/magnetic fields can regulate TGF-β expression, but the results are inconsistent. Shen WW et al. [51] evaluated the effects of a 15 Hz pulsed electromagnetic field (PEMF) on the bone mineral density (BMD) and local factor production in osteoporotic rats. They showed a significant increase in the concentration of TGFβ1 after 8 weeks in the serum of rats exposed to PEMF compared to groups not exposed to the electromagnetic field. The results show that PEMF stimulation can effectively inhibit bone loss and affect bone remodeling by promoting TGF-β1 secretion. Exposure to the electric field induced the proliferation of MC3T3 osteoblast-like cells and increased DNA content and TGF-β1 levels by 39% compared to unstimulated cultures. However, the relationship between induced cell proliferation and TGF-β1 regulation was unclear [52].

Kim H et al. [53] prepared an alginate ferrogel modified with heparin and subjected it to a magnetic field. They obtained the sustained release of TGF-β1 from the ferrogel and applying a magnetic field regulated the release of TGF-β1. Regulation of TGF-β1 production enhanced the chondrogenic differentiation of ATDC5 cells, which were used as a model chondrogenic cell line. Manipulating the release of bioactive molecules, including growth factors from hydrogels under the influence of magnetic field stimulation, may be helpful in drug delivery and tissue regeneration.

Other studies quantifying the in vivo effect of PEMF on the expression of growth factors and metatarsal bone healing time showed significant increases in the placental growth factor (PIGF), brain-derived neurotrophic factor (BDNF), and bone morphogenetic proteins (BMP-7 and BMP-5) that belong to the TGF-β family after PEMF treatment [54].

The results of this study are consistent with previous reports of increased expression of TGF-β factors after PEMF stimulation. Lohman et al. [55] studied the effect of PEMF on osteocytopodone cells. After 2 days of daily 8 h PEMF stimulation, they found a twice as high concentration of TGF-β1 compared to control samples. These studies also showed that the effect of PEMF on TGF-β1 levels was dependent on prostaglandins involving cyclooxygenase-1.

Kimsa-Dudek et al. [56] studied the effect of phenolic acids and a static magnetic field on the expression of TGF-β isoforms in amelanotic melanoma cells. They observed a significant decrease in the concentration of TGF-β1 and TGF-β2 proteins in cells treated with SMF and phenolic acid. The researchers found that a static magnetic field could be used to design new therapeutic strategies for diseases disrupting TGF-β-related signaling cascades. The results of these studies coincide with the results obtained by Obayashi-Ishii concerning the inhibitory effect of static magnetic fields on the secretion of PDGF-AA in human squamous cell carcinoma of the oral cavity, which confirms the phenomenon of exerting different effects of magnetic fields on normal and neoplastic cells.

The results obtained in our study show no relationship between the TGF-β1 concentrations in the study groups exposed to a rotating magnetic field (1 h 25 Hz, 1 h 50 Hz, 3 h 25 Hz, and 3 h 50 Hz) and the control group (*p* = 0.11). The highest concentrations of fibroblast growth factor 1 were observed in the samples placed in the RMF for 3 h at 25 Hz and 1 h at 50 Hz. Therefore, our results are different from those of other scientists. This may be due to high individual variability in TGF-β1 production. In this case, it is also necessary to consider changing the RMF parameters and increasing the study group size.

Insulin-like growth factor 1 promotes tissue growth and development, stimulates cell proliferation, affects lipid, protein, and carbohydrate metabolism, lowers glucose levels, and has anti-aging, anti-inflammatory, anabolic, antioxidant, neuroprotective, and hepatoprotective properties. It protects mitochondria against oxidative damage and reduces intramitochondrial production of free radicals [57]. IGF-1 regulates the anabolic and catabolic pathways in skeletal muscles and their function. In addition to increasing muscle mass, it increases bone density and structure. It is a potent stimulus that improves the healing and regeneration of tendon tissue [58,59,60].

Fitzsimmons RJ et al. [61,62] were the first to report the increased synthesis of growth factors in skeletal tissue in response to electric and electromagnetic fields regulating the IGF-2 receptor in human osteosarcoma cells. Studies have shown an increase in IGF-2 mRNA and protein after cell exposure to the area and have suggested that IGF-2 may partially mediate the proliferation of osteoblast-like cells.

Zhou J et al. [63] also studied the effect of sinusoidal electromagnetic fields (SEMF) on bone formation and resorption in vitro in rat femur tissues. Cultured femoral shaft and metaphyseal tissues were treated with SEMF at 50 Hz, 1.8 mT for 1.5 h daily for 12 days. They studied the effect of treatment on markers of bone formation and resorption and their associated gene expression. They showed that SEMF significantly increased the mRNA expression levels of osteogenic transcription factors OSX, ALP, and IGF-1 in bone tissues. The IGF-1 expression in both metaphyseal and diaphyseal tissues was significantly higher in the SEMF-treated groups than in the controls on days 1 and 5. Increased expression of these osteogenic factors enhances osteoblast differentiation and promotes bone formation.

This finding also aligns with the study by Song CX et al. [64] evaluating the effect of a pulsed magnetic field on the IGF-1 levels in the cerebrospinal fluid of traumatized patients. Patients with brain injury were randomly assigned to the control and magnetic therapy groups, where daily therapy with a pulsed magnetic field of 50 Hz, 20–40 mT, was used for 20 min for the next 14 days. Significantly elevated levels of IGF-1 in the cerebrospinal fluid after 14 days of treatment in the group receiving magnetic therapy were found compared to the level of IGF-1 before and in the control group. The IGF-1 values in the cerebrospinal fluid were not significantly changed in the control group not treated with a magnetic field. The researchers concluded that the pulsed magnetic field might increase the IGF-1 levels in the cerebrospinal fluid of brain injury patients, which may promote recovery of patients’ activities of daily living, suggesting its potential clinical value in treating brain injury.

The results obtained in our study also indicate increased synthesis of IGF-1 in plasma in response to a rotating magnetic field. A significant influence of the rotating magnetic field on IGF-1 concentration was observed (*p* < 0.0001). The highest concentration of insulin-derived growth factor 1 was observed in samples placed in the RMF for 3 h at 25 Hz and in samples placed in the RMF for 1 h at 50 Hz. Thus, the exposure of plasma to the RMF for 3 h at 25 Hz and 1 h at 50 Hz significantly increased the release of IGF-1 and the regenerative potential of platelets. Our results are consistent with other scientists’ research results despite different RMF parameters. In the case of IGF-1, scientists point to the beneficial effect of increased concentration of this factor. However, it should be remembered that the field we use, due to the use of low frequencies and its rotational nature, does not hurt the body like in other studies.

Fibroblast growth factor FGF at the cellular level regulates cellular processes such as proliferation, survival, migration, differentiation, and metabolism. It is a hemostatic agent necessary for tissue maintenance, repair, regeneration, and metabolism. FGF signaling has a cardioprotective effect, is important for proper lung epithelial repair and wound healing, and may increase or decrease tissue fibrosis. FGFs also stimulate angiogenesis, proliferation, and growth of fibroblasts and keratinocytes, creating granulation tissue [65].

Fibroblast growth factor 1 is mainly found in the heart, adrenal glands, brain, pituitary gland, nervous tissues, retina, and bones. FGF-1 is a potent mitogen. It can promote mitosis of cells of mesodermal and ectodermal origin, promote epithelial cell proliferation, and contribute to wound epithelialization. Fibroblast growth factor 2 (FGF-2) may accelerate wound healing by activating vascular endothelial cells, smooth muscles, osteoblasts, and chondrocytes [66].

Tepper OM et al. [67] evaluated the in vitro effect of a pulsed electromagnetic field (PEMF) on angiogenesis, a critical process for successfully healing various tissues. PEMF at 15 Hz increased the degree of endothelial cell tubulation seven-fold and proliferation three-fold in vitro. Screening of angiogenic proteins showed a five-fold increase in FGF-2 and a minor increase in angiogenic factors such as angiopoietin 2, thrombopoietin, and epidermal growth factor EGF. The levels of platelet-derived growth factor PDGF and hepatocyte growth factor HGF were not significantly affected by PEMF exposure compared to the control group.

After showing that PEMF strongly affects endothelial cells in vitro, the researchers investigated whether pulsed electromagnetic fields can stimulate angiogenesis in vivo. A study on transgenic mice showed significantly increased vascular growth and a two-fold increase in FGF-2 in animals treated with PEMF. There was no difference in the TPO, and-2, and EGF growth factors. The scientists concluded that PEMF can increase angiogenesis and FGF-2 secretion. Other studies report a three-fold increase in FGF-2 in human umbilical vein endothelial cell (HUVEC) culture media after 8 h of exposure to a PEMF at 15 Hz. The same study observed a significant increase in FGF-2 release in diabetic mouse wounds exposed to PEMF at 15 Hz compared to the control (155.0 ± 27.0 pg/mL versus 90.0 ± 25.0 pg/mL). The increase in FGF-2 release was the leading cause of accelerated wound healing in diabetic mice after exposure to electromagnetic fields.

Peng L et al. [68], in addition to using PEMF 15 Hz 1.5 mT, also tested the effect of this field with a frequency of 30 Hz 3.0 mT on mice after myocardial infarction. Upregulation of VEGF and VEGFR2 has been reported in mice exposed to PEMF myocardial tissues at 15 Hz and 30 Hz. In addition, a significantly increased level of FGF-2 protein and mRNA, as well as the level of integrin β1 protein, was observed after applying PEMF with a frequency of 30 Hz, indicating a more substantial therapeutic effect.

Our study observed a significant impact of the rotating magnetic field on the concentration of FGF-1 (*p* = 0.0012) in plasma. The highest concentration of FGF-1 was observed in samples placed in the RMF for 3 h at a frequency of 25 Hz and 1 h at 50 Hz. Thus, the exposure of plasma to an RMF for 3 h at 25 Hz or 1 h at 50 Hz significantly increases the release of fibroblast growth factor 1 and the regenerative potential of platelets. These results also confirm those obtained by Tepper OM et al. However, it should be noted that Tepper OM used different electromagnetic field parameters than we did and investigated another isoform of fibroblast growth factor. Moreover, compared to those obtained by other scientists, our results indicate that, when using an RMF, the purpose of its use is very important.

The impact of the RMF on the concentrations of PDGF-BB, TGF-β1, IGF-1, and FGF-1 was also assessed depending on the sex of the volunteers included in the study. A significant difference in IGF-1 concentration (*p* = 0.011) was found between men and women after plasma exposure to the rotating magnetic field.

Higher concentrations of IGF-1 were obtained in the material collected from women. In the case of the other growth factors (PDGF-BB, TGF-β1, and FGF-1), no significant differences were observed between women and men. According to Budkowska et al., the number of platelets in women is slightly higher than in men. These changes are most noticeable in young people. However, despite noticeable differences, these values were not statistically significant. There are no different reference values for platelet counts in men and women [69]. In the study, no statistically significant differences were found in the number of blood platelets between women and men. Additionally, no statistically significant correlations were observed between number of blood platelets and concentration of growth factors. Therefore, it is not easy to interpret whether women’s platelets respond more to RMFs. Further studies are needed to confirm this relationship.

IGF-1 is synthesized mainly in the liver and peripheral tissues; diet, insulin, thyroid, sex hormones, age, pubertal stage, sex, and ethnicity also influence its expression [58,70]. In a study [71] analyzing circulating IGF-1 levels in healthy young adults, IGF-1 levels were negatively correlated with body fat, body mass index (BMI), and total cholesterol and positively correlated with aerobic capacity and muscular endurance parameters. Lower IGF-1 levels have been associated with various pathologies, chronic diseases, inflammation, and malnutrition [72,73]. In the conducted study, a low negative correlation was also observed in all the groups (tested and control groups) between IGF-1 concentration and gender (Rs = −0.232; *p* < 0.05), height (Rs = −0.188; *p* < 0.05) and weight (Rs = −0.257; *p* < 0.05). It was also shown that the increase in weight affected the concentration of IGF-1, causing a decrease in this growth factor by 0.26 ng/mL, respectively, which is consistent with previous reports regarding the effect of weight and BMI on insulin-like factor levels. 

Our study also showed numerous correlations between the concentrations of the tested growth factors (using different frequencies and durations of stay in the RMF). These correlations result from the activation of platelets and the release of these factors from granules. Moreover, such factors as PDGF and TGF have similar effects and cooperate, for example, in the processes of fibrosis or ossification. Therefore, their production is dependent on each other. However, the positive correlation between FGF concentration and the age of the study group (independent of field parameters) is interesting. Hurley et al. showed that a lower level of FGF2 with age may cause impairment in the function of HMDPCs (human progenitor cells derived from mesenchyme) by modulating Wnt-β-catenin signaling, which consequently causes a reduction in bone mass [74]. Our results indicate additional possibilities of using RMFs to prevent bone loss in older people.

## 4. Materials and Methods

### 4.1. Characteristics of the Study Group

The study involved 30 healthy volunteers, 15 women and 15 men aged 21 to 41 (25 ± 5). The health status of the persons qualified for the study was confirmed by determining the basic biochemical parameters in the blood plasma, such as the concentration of cholesterol, HDL, LDL, triglycerides, glucose, albumin, iron, uric acid, creatinine, and total protein, using ready-made reagent kits from BioMaxima S.A., Lublin, Poland, using the EnVision microplate reader (Perkin Elmer, Waltham, MA, USA). A questionnaire study was also carried out regarding height, weight, the occurrence of chronic diseases, taking medications and hormonal contraception, smoking cigarettes, and undergoing surgeries over 6 months. Detailed characteristics of the study group are presented in Table 13, Table 14, Table 15, Table 16 and Table 17. All participants agreed to participate in the study.

### 4.2. Materials

**Figure 7:**
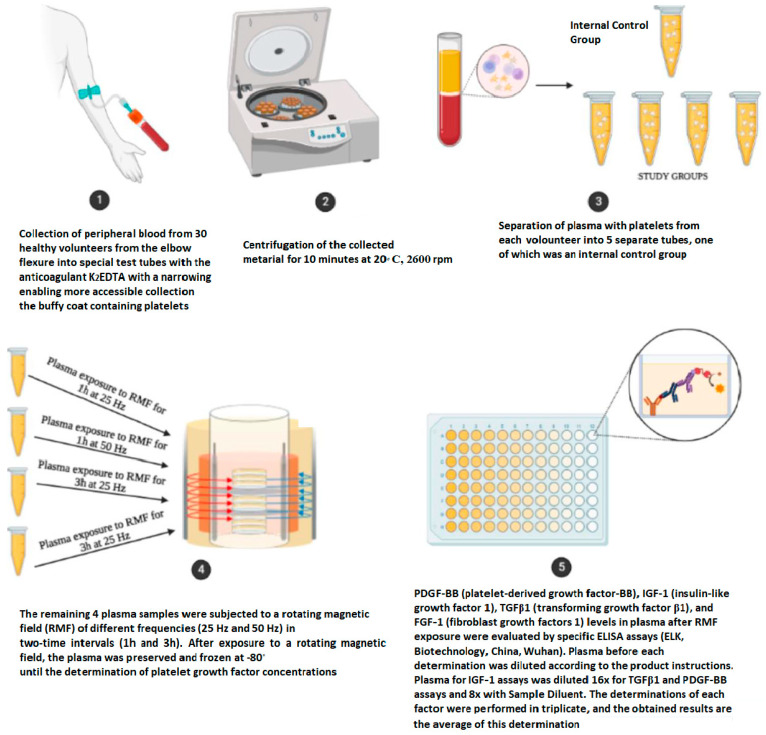
Methods and materials. Graphical representation of methods and materials. Created in BioRender.com.

The material used to determine platelet growth factor concentrations was blood plasma collected once by qualified medical personnel into special test tubes containing the anticoagulant K2EDTA. These tubes were designed with narrowing to facilitate the more accessible collection of the buffy coat, which includes platelets among other components. Then, the collected material was centrifuged for 10 min at 20 °C, 2600 rpm. Plasma from each volunteer was aliquoted into 5 separate tubes, one of which was an internal control group. The remaining plasma samples, immediately after their preparation, were exposed to a rotating magnetic field at varying frequencies (25 Hz and 50 Hz) for two different time intervals (1 h and 3 h). After exposure to an RMF, platelet counts were determined in all samples using a hematology analyzer. Then, the plasma was preserved and frozen at −80 °C until the determination of GF concentrations. According to previous studies, storing platelet-rich plasma at −80 °C does not cause significant changes in platelet count, growth factors’ concentration, the content of 5-HT, and inflammatory effects [75,76,77] (Figure 7).

### 4.3. Experimental Setup

**Figure 8:**
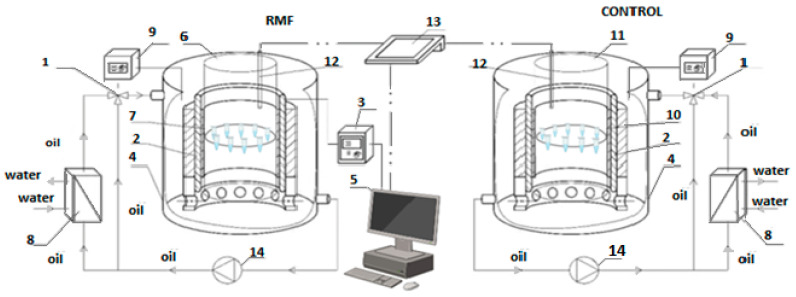
Experimental setup. 1—cooling system; 2—RMF generator; 3—AC transistorized inverter; 4—vessel; 5—personal computer; 6—glass container; 7—probe; 8—heat exchanger; 9—thermostat; 10—control probe; 11—control container; 12—temperature sensor; 13—multifunctional computer meters; 14—circulation pump. Figure based on [78].

Experiments were conducted using an innovative self-designed system featuring a magnetically assisted bioreactor (Figure 8). The rotating magnetic field (RMF) is produced by a set of 3-phase internal windings powered by AC through a phase inverter connected to a PC, enabling control of current parameters. The voltage was maintained at 150 V, with a current frequency of 50 Hz, resulting in a maximum RMF induction of Bmax = 42.64 mT. The RMF generator was positioned inside a cylindrical stainless-steel tank, which shields the field, keeping it confined within the bioreactor. The tank’s center was placed in a second container made of transparent polycarbonate. Probes with bacteria suspension were situated in the inner container filled with water to enhance heat capacity. The inner bath temperature was monitored using a CX-701 multimeter with a Pt-1000 temperature probe.

The study involved calculating averaged values of magnetic induction at various points within the RMF generator, and these values were represented as contour patterns showcasing the spatial distribution of the magnetic field. The analysis, focusing on a horizontal RMF with axial symmetry, revealed that the generator’s magnetic induction remained constant across different angles (φ). The magnetic field lines exhibited horizontal rotation at the frequency of the electric current, resulting in uniform magnetic induction. Notably, maximal induction values were observed at the midpoint of the RMF generator height, measuring 42.64 mT for a frequency of 50 Hz. Full characteristics of the research system used were described earlier by Jabłońska et al. [79].

### 4.4. Methods of Determination of Platelet Growth Factors

The PDGF-BB, TGF-β1, IGF-1, and FGF-1 concentrations in blood plasma were determined by ELISA using a ready-made reagent kit (ELK Biotechnology, Wuhan, China). Plasma before each determination was diluted according to the producent instructions. Plasma for IGF-1 assays was diluted 16×, and, for TGF-β1 and PDGF-BB assays, 8× with Sample Diluent.

For IGF-1, PDGF-BB, and TGF-β1, 100 μL of plasma and standard solutions were added to pre-coated wells with growth factor-specific antibodies. The plate was incubated for 80 min at 37 °C. At the end of incubation, the wells were washed three times with 200 μL of washing buffer. After each rinse, the excess liquids were removed. Then, to each well was added 100 μL of a working solution of biotin-conjugated antibodies specific for a given growth factor and incubated for 50 min at 37 °C. At the end of the incubation, the wells were washed three times with 200 μL of washing buffer and thoroughly dried. In the next step, 100 μL of avidin conjugated to horseradish peroxidase (HRP) was added to each well and incubated for 50 min at 37 °C. At the end of the incubation, the wells were washed five times with 200 μL of washing buffer and thoroughly dried. Then, 90 μL of TMB (tetramethylbenzidine) substrate solution was added to each well and incubated in a dark place for 20 min at 37 °C. After the designated time, 50 μL of sulfuric acid solution was added, stopping the enzyme–substrate reaction. The color change was measured spectrophotometrically at 450 nm using an EnVision microplate reader (Perkin Elmer). The concentrations of GF in the samples were determined by comparing the optical density of the samples with the standard curve, obtained from the determination of different concentrations of standard: PDGF-BB—2000 pg/mL, 1000 pg/mL, 500 pg/mL, 250 pq/mL, 125 pg/mL, 62.5 pg/mL, 31.25 pq/mL, 0 pg/mL; TGF-β1—1000 pg/mL, 500 pg/mL, 250 pq/mL, 125 pg/mL, 62.5 pg/mL, 31.25 pq/mL, 15.63 pg/mL, 0 pg/mL; IGF-1—10 ng/mL, 5 ng/mL, 2.5 ng/mL, 1.25 ng/mL, 0.63 ng/mL, 0.32 ng/mL, 0.16 ng/mL, and 0 ng/mL.

In the case of FGF-1, 40 μL of serum and 50 μL of standard solutions containing biotinylated antibodies were added to the wells pre-coated with human anti-FGF-1 antibodies. Then, 10 μL of biotinylated anti-FGF-1 antibodies and 50 μL of streptavidin-HRP were added to the wells containing samples and standard solutions (except for the blank sample). The plate was incubated at 37 °C for 60 min. After incubation, the wells were washed five times with 300 μL of washing buffer. After each rinse, the excess liquids were removed. In the next step, 50 μL of substrate solution A and 50 μL of substrate solution B were added and incubated in a dark place at 37 °C for 10 min. After the specified time, 50 μL of acidic stop solution was added to terminate the reaction. The color change was measured spectrophotometrically at a wavelength of 450 nm using an EnVision microplate reader (Perkin Elmer). The concentration of fibroblast growth factor 1 in the samples was determined by comparing the optical density of the samples with the standard curve obtained from measurements of different concentrations of the FGF-1 standard (8000 ng/mL, 4000 ng/mL, 2000 ng/mL, 1000 ng/mL, 500 ng/mL, 250 ng/mL, and 0 ng/mL).

The determinations of each factor were performed in triplicate, and the obtained results are the average of this determination.

### 4.5. Statistical Analysis

The obtained results were subjected to statistical analysis. Descriptive statistics (arithmetic mean, lower quartile, upper quartile, median, and standard deviation) of the concentrations of basic biochemical parameters (cholesterol, HDL, LDL, triglycerides, glucose, albumin, iron, uric acid, creatinine, and total protein) were performed to describe the data collected in the surveys (occurrence of chronic diseases, taking medications and hormonal contraception, smoking cigarettes, and undergoing operations in 6 months), and frequency tables (number, cumulative number, and percentage) were prepared. Descriptive statistical analysis (arithmetic mean, median, minimum values, maximum values, interquartile range, and standard deviation) was also applied to the concentrations of platelets and platelet growth factors IGF-1, TGF-β1, PDGF-BB, and FGF-1 depending on the frequency and time of residence of the samples in the RMF. The Shapiro–Wilk test assessed the normality of GFs and PLTs concentration distributions, which showed abnormal distributions of parameters for all variables. The difference in concentrations of platelet growth factors between the study groups (1 h 25 Hz, 3 h 25 Hz, 1 h 50 Hz, and 3 h 50 Hz) exposed to the rotating magnetic field and the control group was checked. A non-parametric Friedman ANOVA, Bonferroni Post-hoc test, and Wilcoxon Signed Ranks test were used for between-group comparisons for IGF-1, TGF-β1, PDGF-BB, and FGF-1 concentrations. The differences in GFs and PLTs concentrations depending on the gender of the study participants were subjected to statistical analysis; the non-parametric U Mann–Whitney test was used. The strength of the correlation between all parameters was determined using Spearman’s rank correlation. The linear multiple regression model was used to determine the multivariate evaluation of the relationships between the examined parameters. The concentrations of PDGF-BB, TGF-β1, IGF-1, and FGF-1 were defined as dependent variables. Group, age, height, and weight were defined as independent variables. Statistical analysis of the obtained results was performed in the R studio/Staticstica PL 13.3 Trial program (TIBCO Software Inc. (2017). Statistica (data analysis software system), version 13. http://statistica.io). *p*-values < 0.05 were considered statistically significant.

## 5. Conclusions

The following conclusions can be drawn based on the obtained results:

1. The rotating magnetic field can cause increased production of platelet growth factors such as IGF-1, PDGF-BB, and FGF-1.
2. Women’s platelets may react more intensely to rotating magnetic fields than men’s due to the higher concentrations of IGF-1 obtained in the material collected from women. However, considering that the concentration of IGF-1 depends on various factors, such as diet, hormones, BMI, total cholesterol, aerobic capacity, body fat, and muscular endurance, increased IGF-1 concentration after RMF exposure might be more personalized and indicative of overall health.
3. Elevated levels of growth factors after exposure to RMF indicate that the rotating magnetic field may activate blood platelets, enhancing their regenerative potential. Achieving higher concentrations of growth factors may regulate the complex wound-healing process, reducing its duration, accelerating tissue reconstruction and regeneration, improving angiogenesis and vascular integrity, and reducing inflammatory states.
4. The results of this study may be used as a new alternative method of platelet activation in the future. However, further research is needed, which will involve using RFM against PRP to check whether platelet-rich plasma can be used to obtain platelet growth factors even more effectively.

## Figures and Tables

**Table 1:** PDGF-BB concentrations [pg/mL] in the control group and the study groups.

Parameter [pg/mL]	n	Min	Max	Median	IQR	Mean	SD
PDGF-BB control	30	905.68	376,680.00	2242.84	2767.37	28,018.03	92,363.65
PDGF-BB 1 h 25 Hz	30	813.32	352,680.00	2895.97	2986.43	15,668.34	63,870.67
PDGF-BB 1 h 50 Hz	30	1013.44	32,000.00	2149.49	2397.97	4744.49	7595.15
PDGF-BB 3 h 25 Hz	30	759.44	324,280.00	2402.75	2395.30	15,088.64	58,776.32
PDGF-BB 3 h 50 Hz	30	1054.49	12,060.80	2524.85	2425.92	3522.31	2880.52

PDGF-BB—platelet-derived growth factor BB; n—number of people; Min—minimum values; Max—maximum values; IQR—interquartile range; SD—standard deviation.

**Table 2:** TGF-β1 concentrations [pg/mL] in the control group and in the study groups.

Parameter [pg/mL]	n	Min	Max	Median	IQR	Mean	SD
TGF-β1 control	30	541.61	11,319.84	3058.98	2790.61	3780.59	2454.98
TGF-β1 1 h 25 Hz	30	1299.46	13,062.08	3248.65	2572.69	4392.48	2914.41
TGF-β1 1 h 50 Hz	30	9.98	2,227,244.10	3486.75	2107.59	78,269.29	405,886.01
TGF-β1 3 h 25 Hz	30	2203.43	17,337.60	3441.18	2649.07	4646.24	3142.56
TGF-β1 3 h 50 Hz	30	2102.54	11,973.61	3331.38	1771.63	4114.96	2330.23

TGF-β1—transforming growth factor beta 1; n—number of subjects; Min—minimum values; Max—maximum values; IQR—interquartile range; SD—standard deviation.

**Table 3:** IGF-1 concentrations [ng/mL] in the control group and in the study groups.

Parameter [ng/mL]	n	Min	Max	Median	IQR	Mean	SD
IGF-1 control	30	0.37	1063.52	3.66	6.18	77.34	247.61
IGF-1 1 h 25 Hz	30	0.55	261.64	3.56	6.94	19.59	54.82
IGF-1 1 h 50 Hz	30	0.69	180.98	6.91	9.08	17.19	42.34
IGF-1 3 h 25 Hz	30	0.99	462.99	8.74	12.87	34.40	94.10
IGF-1 3 h 50 Hz	30	0.92	15,752.00	4.62	11.11	1055.52	3991.58

IGF-1—insulin-derived growth factor 1; n—number of people; Min—minimum values; Max—maximum values; IQR—interquartile range; SD—standard deviation.

**Table 4:** FGF-1 concentrations [ng/mL] in the control group and the study groups.

Parameter [ng/mL]	n	Min	Max	Median	IQR	Mean	SD
FGF-1 control	30	654.30	5494.30	1165.65	655.09	1431.96	1052.94
FGF-1 1 h 25 Hz	30	704.07	5503.60	1295.40	794.07	1596.83	1062.08
FGF-1 1 h 50 Hz	30	703.07	10,037.00	1357.35	1000.09	1831.36	1835.66
FGF-1 3 h 25 Hz	30	718.03	8895.70	1378.20	1285.93	1902.93	1805.91
FGF-1 3 h 50 Hz	30	653.30	14,897.00	1336.75	996.89	2579.48	1900.86

FGF-1—fibroblast growth factor 1; n—number of people; Min—minimum values; Max—maximum values; IQR—interquartile range; SD—standard deviation.

**Table 5:** Platelet concentrations [μL] in the samples for all cases, women and men.

Group	n	Mean	Median	SD	Min	Max
For all cases	30	210,533.3	196,500	75,773.13	132,000	441,000
Women	15	187,500	179,000	52,033.32	132,000	343,000
Men	15	236,857.1	208,000	91,021.43	137,000	441,000

n—number of people; Min—minimum values; Max—maximum values; median; SD—standard deviation.

**Table 6:** Spearman’s rank correlation coefficients between the concentrations of IGF-1, TGF-β1, PDGF-BB, and FGF-1 in plasma and the tested parameters in all study groups and in the control group (Rs; *p*).

IGF-1 [ng/mL]	TGF-β1 [pg/mL]	PDGF-BB [pg/mL]	FGF-1 [ng/mL]
Sex (Rs = −0.232; *p* = 0.004)	Age (Rs = 0.213; *p =* 0.009)	Age (Rs = 0.212; *p =* 0.009)	Age (Rs = 0.515; *p* < 0.001)
Height (Rs = −0.188; *p =* 0.021)	Weigt (Rs = −0.270; *p =* 0.001)	Height (Rs = −0.186; *p =* 0.023)	Height (Rs = −0.219; *p =* 0.007)
Weight (Rs = −0.257; *p =* 0.001)	PDGF-BB (Rs = 0.648; *p* < 0.001)	Weight (Rs = −0.180; *p =* 0.027)	IGF-1 (Rs = 0.464; *p =* 0.000)
PDGF-BB (Rs = 0.234; *p =* 0.004)	FGF-1 (Rs = 0.317; *p =* 0.000)	IGF-1 (Rs = 0.234; *p =* 0.004)	TGF-β1 (Rs = 0.317; *p =* 0.000)
FGF-1 (Rs = 0.464; *p =* 0.000)	-	TGF-β1 (Rs = 0.648; *p =* 0.000)	PDGF-BB (Rs = 0.564; *p =* 0.000)
-	-	FGF-1 (Rs = 0.564; *p =* 0.000)	-

Rs—Spearman’s rank correlation coefficients; *p*-value of significance coefficient; PDGF-BB—platelet-derived growth factor BB; TGF-β1—transforming growth factor beta 1; IGF-1—insulin-like growth factor 1; FGF-1—fibroblast growth factor 1.

**Table 7:** Spearman’s rank correlation coefficients between plasma concentrations of IGF-1, TGF-β1, PDGF-BB, FGF-1, and the tested parameters in the control group (Rs; *p*).

IGF-1 [ng/mL]	TGF-β1 [pg/mL]	PDGF-BB [pg/mL]	FGF-1 [ng/mL]
Weight (Rs = −0.430; *p* = 0.018)	PDGF-BB (Rs = 0.696; *p* = 0.000)	TGF-β1 (Rs = 0.696; *p* = 0.000)	Age (Rs = 0.498; *p* = 0.005)
-	FGF-1 (Rs = 0.415; *p* = 0.022)	FGF-1 (Rs = 0.600; *p* = 0.001)	Total protein (Rs = 0.445; *p* = 0.014)
-	-	-	TGF-β1 (Rs = 0.415; *p* = 0.022)
-	-	-	PDGF-BB (Rs = 0.600; *p* = 0.001)

Rs—Spearman’s rank correlation coefficients; *p*-value of significance coefficient; PDGF-BB—platelet-derived growth factor BB; TGF-β1—transforming growth factor beta 1; IGF-1—insulin-like growth factor 1; FGF-1—fibroblast growth factor 1.

**Table 8:** Spearman’s rank correlation coefficients between the concentrations of IGF-1, TGF-β1, PDGF-BB, and FGF-1 in plasma and the tested parameters in the study group 1 h 25 Hz (Rs; *p*).

IGF-1 [ng/mL]	TGF-β1 [pg/mL]	PDGF-BB [pg/mL]	FGF-1 [ng/mL]
FGF-1 (Rs = 0.426; *p* = 0.019)	PDGF-BB (Rs = 0.450; *p* = 0.013)	TGF-β1 (Rs = 0.450; *p* = 0.013)	Age (Rs = 0.475; *p* = 0.008)
-	-	FGF-1 (Rs = 0.657; *p* = 0.000)	IGF-1 (Rs = 0.426; *p* = 0.019)
-	-	-	PDGF-BB (Rs = 0.657; *p* = 0.000)

Rs—Spearman’s rank correlation coefficients; *p*-value of significance coefficient; PDGF-BB—platelet-derived growth factor BB; TGF-β1—transforming growth factor beta 1; IGF-1—insulin-like growth factor 1; FGF-1—fibroblast growth factor 1.

**Table 9:** Spearman’s rank correlation coefficients between plasma concentrations of IGF-1, TGF-β1, PDGF-BB, FGF-1, and the tested parameters in the study group 1 h 50 Hz (Rs; *p*).

IGF-1 [ng/mL]	TGF-β1 [pg/mL]	PDGF-BB [pg/mL]	FGF-1 [ng/mL]
FGF-1 (Rs = 0.600; *p* = 0.001)	PDGF-BB (Rs = 0.714; *p* = 0.000)	TGF-β1 (Rs = 0.714; *p* = 0.000)	Age (Rs = 0.497; *p* = 0.005)
-	-	FGF-1 (Rs = 0.600; *p* = 0.001)	IGF-1 (Rs = 0.600; *p* = 0.001)
-	-	-	PDGF-BB (Rs = 0.600; *p* = 0.001)

Rs—Spearman’s rank correlation coefficients; *p*-value of significance coefficient; PDGF-BB—platelet-derived growth factor BB; TGF-β1—transforming growth factor beta 1; IGF-1—insulin-like growth factor 1; FGF-1—fibroblast growth factor 1.

**Table 10:** Spearman’s rank correlation coefficients between plasma concentrations of IGF-1, TGF-β1, PDGF-BB, FGF-1, and the tested parameters in the study group 3 h 25 Hz (Rs; *p*).

IGF-1 [ng/mL]	TGF-β1 [pg/mL]	PDGF-BB [pg/mL]	FGF-1 [ng/mL]
FGF-1 (Rs = 0.592; *p* = 0.001)	PDGF-BB (Rs = 0.519; *p* = 0.003)	TGF-β1 (Rs = 0.519; *p* = 0.003)	Age (Rs = 0545; *p* = 0.002)
-	-	FGF-1 (Rs = 0.526; *p* = 0.003)	IGF-1 (Rs = 0.592; *p* = 0.001)
-	-	-	PDGF-BB (Rs = 0.526; *p* = 0.003)

Rs—Spearman’s rank correlation coefficients; *p*-value of significance coefficient; PDGF-BB—platelet-derived growth factor BB; TGF-β1—transforming growth factor beta 1; IGF-1—insulin-like growth factor 1; FGF-1—fibroblast growth factor 1.

**Table 11:** Spearman’s rank correlation coefficients between the concentrations of IGF-1, TGF-β1, PDGF-BB, and FGF-1 in plasma and the tested parameters in the study group 3 h 50 Hz (Rs; *p*).

IGF-1 [ng/mL]	TGF-β1 [pg/mL]	PDGF-BB [pg/mL]	FGF-1 [ng/mL]
FGF-1 (Rs = 0.564; *p* = 0.001)	PDGF-BB (Rs = 0.818; *p* = 0.000)	TGF-β1 (Rs = 0.818; *p* = 0.000)	Age (Rs = 0.518; *p* = 0.003)
-	FGF-1 (Rs = 0.387; *p* = 0.035)	FGF-1 (Rs = 0.494; *p* = 0.006)	IGF-1 (Rs = 0.564; *p* = 0.001)
-	-	-	TGF-β1 (Rs = 0.387; *p* = 0.035)
-	-	-	PDGF-BB (Rs = 0.494; *p* = 0.006)

Rs—Spearman’s rank correlation coefficients; *p*-value of significance coefficient; PDGF-BB—platelet-derived growth factor BB; TGF-β1—transforming growth factor beta 1; IGF-1—insulin-like growth factor 1; FGF-1—fibroblast growth factor 1.

**Table 12:** Multivariate regression analysis of the influence of the tested parameters on the concentration values of platelet growth factors PDGF-BB, TGF-β1, IGF-1, and FGF-1.

Dependent Variable	Independent Variable	β	R^2^	*p*	*p* for Model	F
PDGF-BB [pg/mL]	Group	−0.15	0.12	NS	0.040	2.55
	Age	0.19		0.03		
	High	−0.07		0.56		
	Weight	0.06		0.62		
TGF-β1 [pg/mL]	Group	0.06	0.004	NS	NS	0.74
	Age	0.06		NS		
	High	0.19		NS		
	Weight	−0.09		NS		
IGF-1 [ng/mL]	Group	0.15	0.02	NS	0.049	2.45
	Age	−0.08		NS		
	High	0.14		NS		
	Weight	−0.26		0.03		
FGF-1 [ng/mL]	Group	0.095	0.18	NS	<0.001	12.04
	Age	0.443		<0.001		
	High	−0.159		NS		
	Weight	0.316		0.004		

β—standardized coefficient in the regression equation; R^2^—coefficient of determination; *p*-value of significance coefficient; NS—statistically insignificant; PDGF-BB—platelet-derived growth factor BB; TGF-β1—transforming growth factor beta 1; IGF-1—insulin-like growth factor 1; FGF-1—fibroblast growth factor 1.

**Table 13:** Characteristics of the study group in terms of biochemical parameters.

Parameter	Median	Mean	Lower Quartile	Upper Quartile	SD
Cholesterol [mg/dL]	154.42	158.75	136.28	184.51	28.90
HDL [mg/dL]	40.23	42.47	33.86	50.18	14.17
LDL [mg/dL]	94.06	97.42	76.49	119.90	28.60
Glucose [mg/dL]	97.33	97.94	92.00	101.78	7.63
Triglycerides [mg/dL]	132.84	135.92	114.43	153.23	25.96
Albumin [g/dL]	3.88	3.93	3.74	4.18	0.36
Iron [μg/dL]	70.09	68.16	50.47	82.24	19.49
Total protein [g/dL]	6.30	6.35	6.00	6.63	0.46
Uric acid [mg/dL]	4.47	4.51	3.68	5.26	1.10
Creatinine [mg/dL]	0.83	0.86	0.76	1.02	0.19

SD—standard deviation; HDL—high-density lipoprotein; LDL—low-density lipoprotein.

**Table 14:** Characteristics of the study group in terms of the occurrence of chronic diseases.

Prevalence of Chronic Diseases	Number	Percent
No	23	76.67
Epilepsy	1	3.33
Asthma	2	6.67
Hashimoto’s	3	10.00
JIA	1	3.33

JIA—juvenile idiopathic arthritis.

**Table 15:** Characteristics of the study group in terms of taking medications.

Medications Taken	Number	Percent
No	23	76.67
Levetivacetam, Lamotigrinum	1	3.33
Budesonide, formoterol fumarate dihydrate	1	3.33
levothyroxine	1	3.33
levothyroxine, desogestrel	1	3.33
Bisoprololi fumarans, levothyroxine	1	3.33
Dienogest	1	3.33
Methotrexate	1	3.33

**Table 16:** Characteristics of the study group including smoking and taking hormonal contraception.

Factor	Class	Number	Percent
Smoking	Yes	24	80.00
	No	6	20.00
	Unanswered	0	0.00
Taking hormonal contraception	Yes	14	46.67
	No	3	10.00
	Unanswered	13	43.33

**Table 17:** Characteristics of the study group in terms of operations performed within 6 months.

Past Operations within 6 Months	Number	Percent
No	27	90.00
Acute pancreatitis	1	3.33
Jaw surgery	1	3.33
Bone marrow donor	1	3.33

## Data Availability

All data generated or analyzed during this study are included in this published article.
